# Echovirus 30 Induced Neuronal Cell Death through TRIO-RhoA Signaling Activation

**DOI:** 10.1371/journal.pone.0036656

**Published:** 2012-05-07

**Authors:** June-Woo Lee, Sang-Gu Yeo, Byung-Hak Kang, Hoe-Kyu Lee, Jin-Won Kim, Sun-Hwa Lee, Ki-Sang Kim, Doo-Sung Cheon

**Affiliations:** Division of Enteric and Hepatitis Viruses, Center for Infectious Diseases, National Institutes of Health, Osong, Korea; National University of Singapore, Singapore

## Abstract

**Background:**

Echovirus 30 (Echo30) is one of the most frequently identified human enteroviruses (EVs) causing aseptic meningitis and encephalitis. However the mechanism underlying the pathogenesis of Echo30 infection with significant clinical outcomes is not completely understood. The aim of this investigation is to illustrate molecular pathologic alteration in neuronal cells induced by Echo30 infection using clinical isolate from young patient with neurologic involvement.

**Methodology/Principal Findings:**

To characterize the neuronal cellular response to Echo30 infection, we performed a proteomic analysis based on two-dimensional gel electrophoresis (2-DE) and MALDI-TOF/TOF Mass Spectrophotometric (MS) analysis. We identified significant alteration of several protein expression levels in Echo30-infected SK-N-SH cells. Among these proteins, we focused on an outstanding up-regulation of Triple functional domain (TRIO) in Echo30-infected SK-N-SH cells. Generally, TRIO acts as a key component in the regulation of axon guidance and cell migration. In this study, we determined that TRIO plays a role in the novel pathways in Echo30 induced neuronal cell death.

**Conclusions/Significance:**

Our finding shows that TRIO plays a critical role in neuronal cell death by Echo30 infection. Echo30 infection activates TRIO-guanine nucleotide exchange factor (GEF) domains (GEFD2) and RhoA signaling in turn. These results suggest that Echo30 infection induced neuronal cell death by activation of the TRIO-RhoA signaling. We expect the regulation of TRIO-RhoA signaling may represent a new therapeutic approach in treating aseptic meningitis and encephalitis induced by Echo30.

## Introduction

Echovirus 30 (Echo30) is a single-strand positive sense RNA virus that belongs to the genus Enterovirus of the Picornaviridae family [1], [2], [3]. The common transmission routes may be direct, such as by fecal-oral and respiratory spread or indirect, such as by fomites and contaminated water [4]. Primary infection with an Echovirus leads to viral replication in the tissue around the gastrointestinal tract, followed by a transient viremia and sometimes migration into other tissues [5].

Enteroviruses (EVs) are the major causative agents of the central nervous system (CNS) viral infection [6], [7], [8], [9]. The CNS involvement in neonates may not be accompanied by overt signs of meningeal inflammation [6]. The CNS disease in newborns caused by EVs may also progress to meningoencephalitis with the appearance of seizures and focal neurological deficits. Recently, Leong WF et al. have reported that transcriptomic and proteomic analyses of rhabdomyosarcoma cells revealed differential cellular gene expressions in response to Enterovirus 71 (EV71) infection [10]. In 2008, Echo30 associated with an aseptic meningitis outbreak occurred in Korea [1]. It was the first investigation of the molecular characteristics of Echo30 strains associated with aseptic meningitis outbreak in Korea and resulted in a sharp increase in hospitalizations due to neurovirulent symptoms was observed [1]. As such, it is very important to understand the neurovirulent mechanism of Echo30.

In this study, we characterized the neuronal cellular response to Echo30 infection and performed 2-D gel electrophoresis. As a result, we found changes in the expressions of 12 proteins, such as Protein disulfide isomerase-related protein 5 (PDI 5), Tubulin alpha 1a (TUBA 1A) and triple functional domain (TRIO) protein. Interestingly, Echo30 infection to the neuronal cells increases the protein expression of the TRIO.

TRIO proteins are expressed ubiquitously in various tissues including the central nervous system [11], [12], [13]. TRIO contains two functional guanine nucleotide exchange factor (GEF) domains [13], GEFD1 and GEFD2, which specifically activate the Rac1 and RhoA, respectively [13], [14]. The GEFDs for Rho-GTPases activate the GTPases by accelerating the GDP/GTP exchange [15], [16]. TRIO plays an important role in neuronal cell migration and axon guidance via a GEFD1-dependent process [17], [18], [19], [20], [21], [22], [23]. GEFD2 of TRIO acts specifically on RhoA [14], [24], [25]. We presumed GEFD2-mediated activation of RhoA and RhoA targeted signaling, such as Rho-associated protein kinase (ROCK) and myosin-light chain (MLC) which are involved in Echo30 induced neuronal cell death. RhoA signaling is known to promote actin stress fiber formation [26], [27]. We predicted GEFD2 leading to actin stress fiber formation via activation of RhoA signaling. Actin stress fiber formation is particularly important in neuronal cells and Echo30 infection may contribute to barrier dysfunction. Actin stress fiber formation increases the free radical nitric oxide level through the regulation of the endothelial nitric oxide synthase (eNOS) in neuronal cells [28], [29].

**Figure 1:**
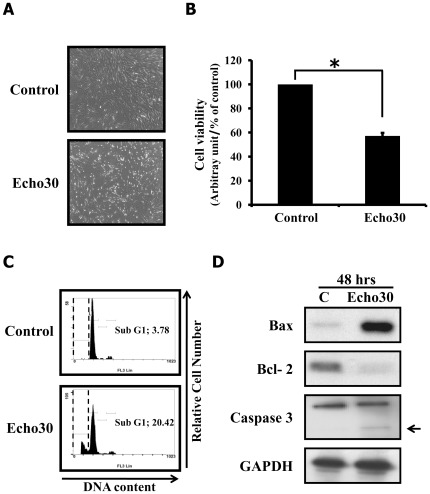
Echo30 infection induced neuronal cell death. Human neuroblastoma SK-N-SH cells at 80% confluence were infected with Echo30 with 2% FBS contained MEM at 48 hrs. (A) The effect of Echo30 infection on SK-N-SH cells. Echo30 infected SK-N-SH cells significantly increase cytopathic effect. (B) Measurement of cell viability by WST-1 assay. The viability of SK-N-SH cells was decreased by Echo30 infection (mean±S.E.M, *p<0.05). (C) Measurement of sub-G1 phase using flow cytometry analysis. Echo30 infection increase sub-G1 phase at cell cycle distribution. (D) Expression of apoptotic proteins using immunoblot analysis. The expression level of pro-apoptotic protein was increased by Echo30 infection. All data are representative of three independent experiments.

In this study, the activations of TrioGEFD2 and RhoA were observed to play important roles in Echo30 induced neuronal cell death. Our finding suggests that the TRIO protein is a new therapeutic target for Echo30 induced neuronal diseases.

**Figure 2:**
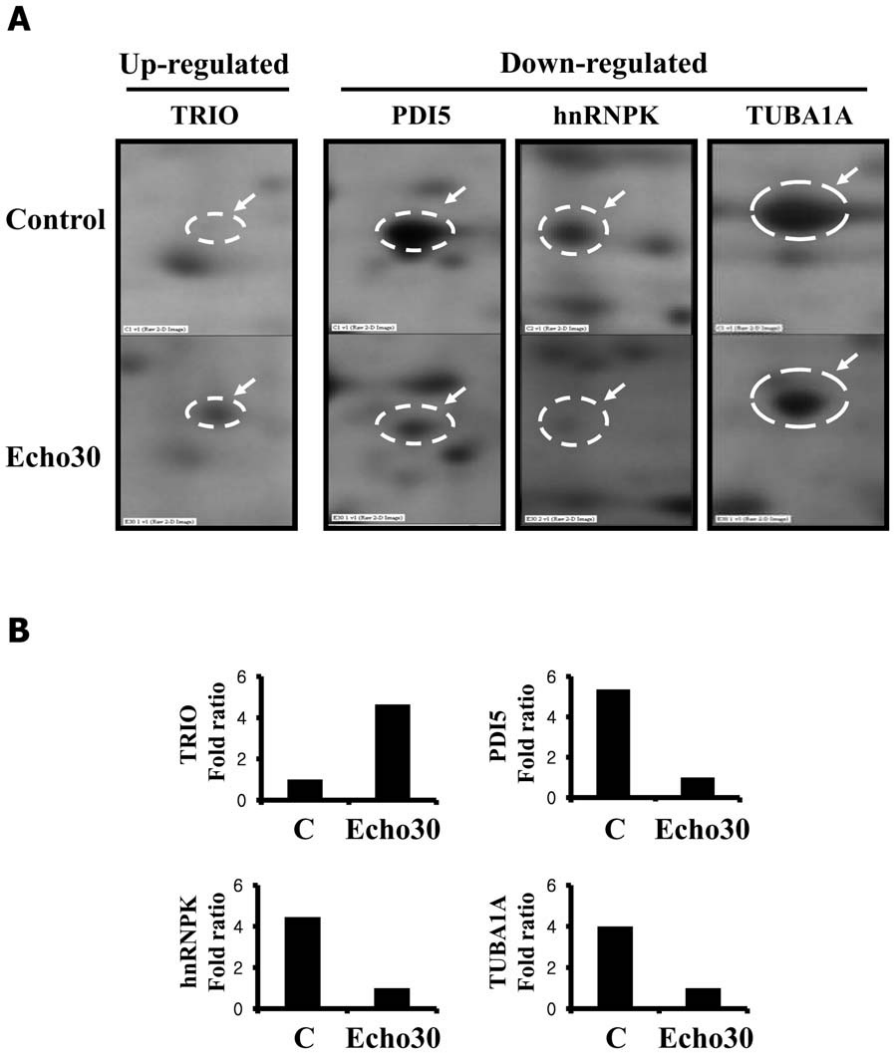
Protein identification of differentially expressed cellular proteins in Echo30-infected SK-N-SH cells at 48 hrs by two-dimensional gel electrophoresis (2-DE). (A) The arrows indicated the protein spots identified as differentially regulated at greater than four-fold changes (*p*<0.05). (B) The spot density was represented relative to the control (non-infected SK-N-SH cells).

## Results

### Echovirus 30 induces neuronal cell death

We first identified the effects of Echo30 infection on human neuroblastoma cell line SK-N-SH. After SK-N-SH cells grew approximately 80% confluent, Echo30 (M.O.I. of 1) was subsequently seeded for 48 hrs in MEM medium containing 2% FBS. As a result, Echo30-infected SK-N-SH cells showed a cytopathic effect (Fig. 1A) and a significant decrease of cell viability (Fig. 1B). In addition, the distribution of sub-G1 phase was increased by almost 16% (Fig. 1C). Furthermore, Echo30 infection induced significant pro-apoptotic protein Bax expression and activated caspase 3 (Fig. 1D). Echo30 infection also reduced anti-apoptotic protein Bcl-2 expression (Fig. 1D). These results suggest that Echo30 infection induces SK-N-SH cell death. However, these events related to the molecular mechanism remain unclear.

### Protein identification of differentially expressed proteins in Echovirus 30 infected neuronal cells

Next, we characterized the pathophysiology of Echo30 infection and neuronal host cellular responses. We performed 2-DE proteomic analysis to probe into the cellular protein expression changes during the infection process. We obtained approximately 1,500 spots using 2-DE proteomic analyses (data not shown). A total of 20 protein spots with a threshold greater than 2-fold were excised from these 2-DE gels and in-gel trypsin digestion and subsequently, MALDI-TOF and MALDI-TOF/TOF identification were carried out. Two-dimensional proteomic maps showed differences in the expression of 12 proteins (Table 1), including down-regulation of Protein disulfide isomerase-related protein 5 (PDI5), Tubulin, alpha 1a (TUBA1A), Tubulin alpha 6 (TUBA6), Uracil DNA glycosylase (UNG), Lamin B1 (LMNB1), Heterogeneous nuclear ribonucleoprotein K (hnRNPK), Ribosomal protein, large P0 (RPLP0), ATP-dependent DNA helicase and up-regulation of Switch-associated protein 70 (SWAP70), Chain A mutant of Annexin VI, and Triple functional domain (TRIO) in Echo30-infected SK-N-SH cells. Most of the altered cellular proteins appeared to have roles in revealing the viral pathogenesis and especially TRIO protein was associated with neuronal cell damage (Fig. 2).

**Table 1:** Identification of differentially expressed proteins in Echo30-infected SK-N-SH cells at 48 hrs by MALDI-TOF.

Spot no.	Protein name	Accession no.	Mw(kDa) and pi	MS method	% coverage	Fold-change
1410	Protein disulfide isomerase-related protein 5	AAB50217	49.7/4.65	MS, MS/MS	35	−5.36
1512	Tubulin alpha 1a (TUBA1A)	NP006000	53.9/4.68	MS, MS/MS	43	−4
2002	Uracil DNA glycosylase (UNG)	CAG46474	26.9/4.79	MS	9	−2.94
2215	Tubulin alpha 6 (TUBA6)	NP116093	39.6/4.98	MS	21	−2.79
2401	Lamin B1 (LMNB 1)	AAH76178	46.9/4.77	MS	30	−4.66
3610	Heterogeneous nuclear ribonucleoprotein K	CAA51267	61.2/5.11	MS, MS/MS	23	−4.45
4216	Ribosomal protein large PO (RPLPO)	AAH01127	39.9/5.44	MS	33	−6.3
4221	Chain A mutant of Annexin VI	pdb1M91	39.5/5.30	MS	19	n/a^+^
5308	Switch-associated protein 70 (SWAP70)	AAF24486	42.2/5.60	MS	9	2.32
5414	Triple functional domain (TRIO)	AAH17268	49.1/5.56	MS	15	4.72
5706	ATP-dependent DNA helicase	NP066964	62.6/5.56	MS	13	4.64
6213	Blue cone opsin	AAL31362	38.6/7.44	MS	16	n/a^+^

* n/a^+^: Detected only in SK-N-SH cells infected with Echo30.

### Echovirus 30 infection induces TRIO-RhoA signaling activation

**Figure 3:**
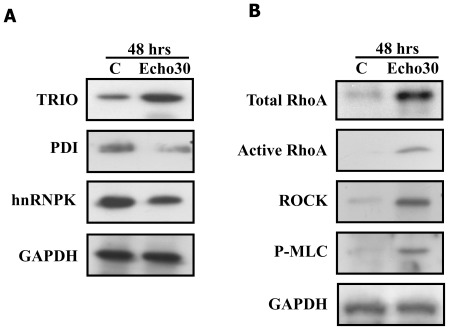
Echo30 infection induced TRIO-RhoA signaling activation in neuronal cell. Human neuroblastoma SK-N-SH cells at 80% confluence were infected with Echo30 with 2% FBS contained MEM at 48 hrs. (A) Confirmation results of 2-DE using immunoblot blot analysis. TRIO protein expression was significantly increased in Echo30-infected SK-N-SH cells. (B) Immnoblot analysis for RhoA signaling. Echo30 infection induced activation of RhoA and RhoA targeted signaling in SK-N-SH cell. All data are representative of three independent experiments.

To elucidate the role of TRIO proteins involved in neuronal cell death induced by Echo30 infection, we determined TRIO protein expression level using immunoblot analysis. As shown in Fig. 3A, TRIO protein expression was significantly increased in Echo30-infected SK-N-SH cells concomitant with reduction of PDI and hnRNPK levels, whereas GAPDH expression was unaffected. Furthermore, Echo30 infection induced the activation of RhoA and RhoA targeted signaling, such as Rho-associated protein kinase (ROCK) and phosphorylation of myosin-light chain (p-MLC) (Fig. 3B). TrioGEFD2 is known as upstream of RhoA and RhoA targeted signaling [24], [25]. Activation of RhoA and RhoA targeted signaling promotes actin stress fiber formation [26], [27], leading to phosphorylation of MLC and activation of ROCK [26], [27]. These data suggest that Echo30 infection induces actin stress fiber formation through the activation of TrioGEFD2-RhoA signaling and finally results in neuronal cell death.

### Echovirus 30 infection enhances NO production through TRIO-RhoA signaling activation

**Figure 4:**
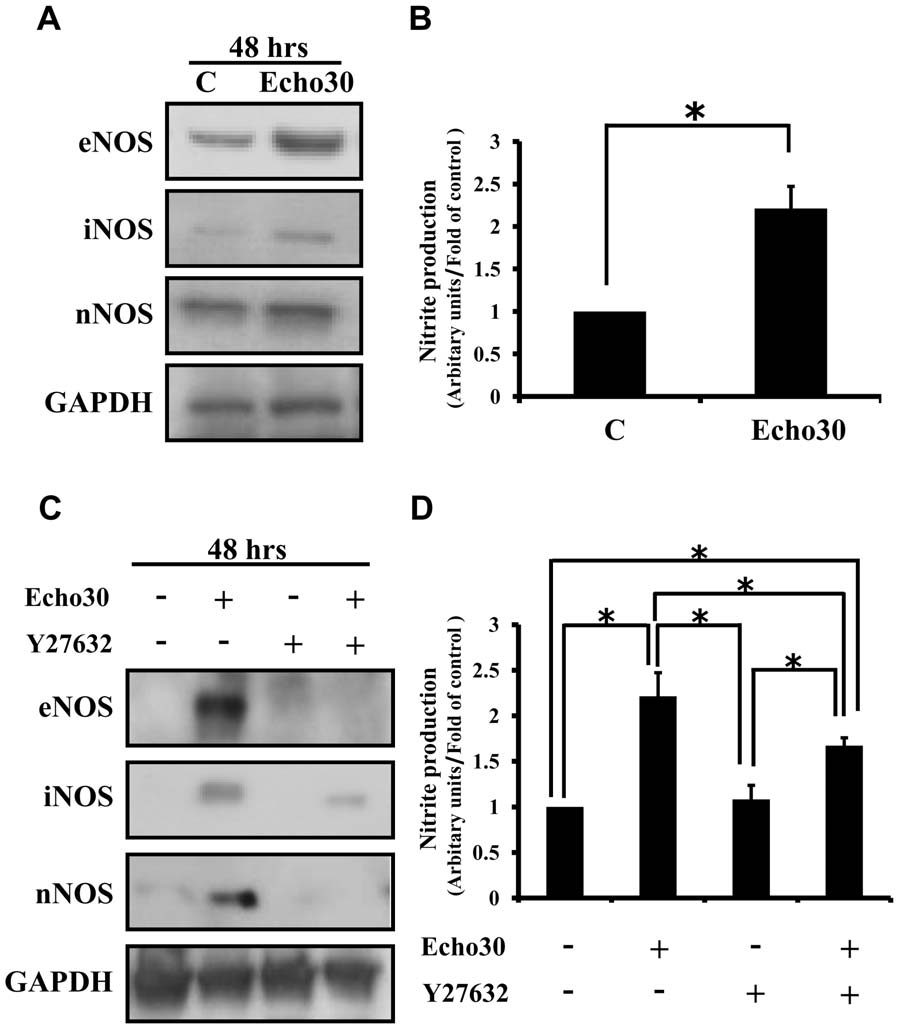
Echo30 infection enhanced NO production through TRIO-RhoA signaling activation in neuronal cells. (A) Echo30-infected SK-N-SH cells increase NOS family (eNOS, iNOS, nNOS) protein levels using immunoblot analysis. Echo30 infection enhanced NOS family protein levels. (B) Measurement of cellular NO production by griess reagent system. Echo30-infected SK-N-SH cells significantly increase cellular NO level (mean±S.E.M, *p<0.05). (C) The effect of Rho kinase inhibitor Y27632 on NOS family induction by Echo30 infection. Pre-treatment of cells with the Y27632 prevent NOS family induction by Echo30 infection. (D) The effect of Y27632 on NO production by Echo30 infection. Pre-treatment of cells with the Y27632 blocked NO production by Echo30 infection (mean±S.E.M, *p<0.05). All data are representative of three independent experiments.

We identified Echo30 infection lead to increase production of nitric oxide (NO). Based on this result, we investigated the relationship between NO production by Echo30 infection in neuronal cells and TrioGEFD2 activation. As shown in Fig. 4A, Echo30-infected SK-N-SH cell increased endothelial nitric oxide synthase (eNOS), inducible nitric oxide synthase (iNOS), and neural nitric oxide synthase (nNOS) protein level. In addition, Echo30 infection increased cellular nitric oxide (NO) level (Fig. 4B). Echo30 infection induced NO production in neuronal cells via actin stress fiber formation. RhoA acts as a regulator of the actin stress fiber formation and eNOS expression [28], [29], [30]. In addition, our results show that Echo30 infection induces not only eNOS expression but also iNOS and nNOS expressions in SK-N-SH cells. Hence, we suggest that activation of TrioGEFD2 promotes NO production via activation of RhoA signaling and induction of NOS family in Echo30-infected neuronal cells.

Next, to examine the relationship between NO production and TrioGEFD2 signaling activation and neuronal cell death by Echo30 infection, we investigated the effects of Y27632, the Rho kinase (ROCK) inhibitor, NO production, and TrioGEFD2 signaling in Echo30-infected SK-N-SH cells. As shown in Fig. 4C, D, pretreatment of Y27632 significantly decreased NOS family induction (Fig. 4C) and NO production (Fig. 4D) in Echo30-infected SK-N-SH cells. These data suggest that Echo30 infection induced neuronal cell death through promoting cellular NO level via activation of TrioGEFD2 and RhoA signaling.

### Rho kinase inhibitor prevents Echovirus 30 induces neuronal cell death

**Figure 5:**
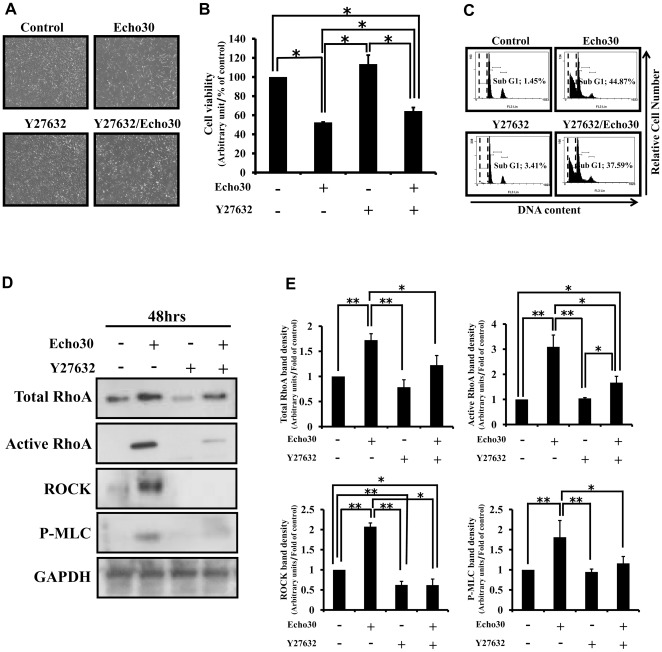
Rho kinase inhibitor prevented neuronal cell death by Echo30 infection. (A) The effect of Y27632 on the cytopathic effect by Echo30 infection. The pretreatment of Y27632 inhibited Echo30 infection-inducing cytopathic effect in SK-N-SH cells. (B) The effect of Y27632 on cell viability by Echo30 infection determined by WST-1 assay. Y27632 inhibited Echo30 infection-inducing SK-N-SH cell death. (mean±S.E.M, *p<0.05). (C) The effect of Y27632 on cell cycle analysis by Echo30 infection using FACS analysis. Y27632 inhibited increase of sub-G1 phase by Echo30 infection in SK-N-SH cells. (D) The effect of Y27632 on RhoA signaling by Echo30 infection using immunoblot analysis. Y27632 prevented Echo30 infection induced activation of RhoA signaling. (E) Graphical representation of densitometric analysis of the immnoblot bands for TRIO-RhoA signaling (mean±S.E.M, **p<0.05, *p<0.1). All data are representative of three independent experiments.

**Figure 6:**
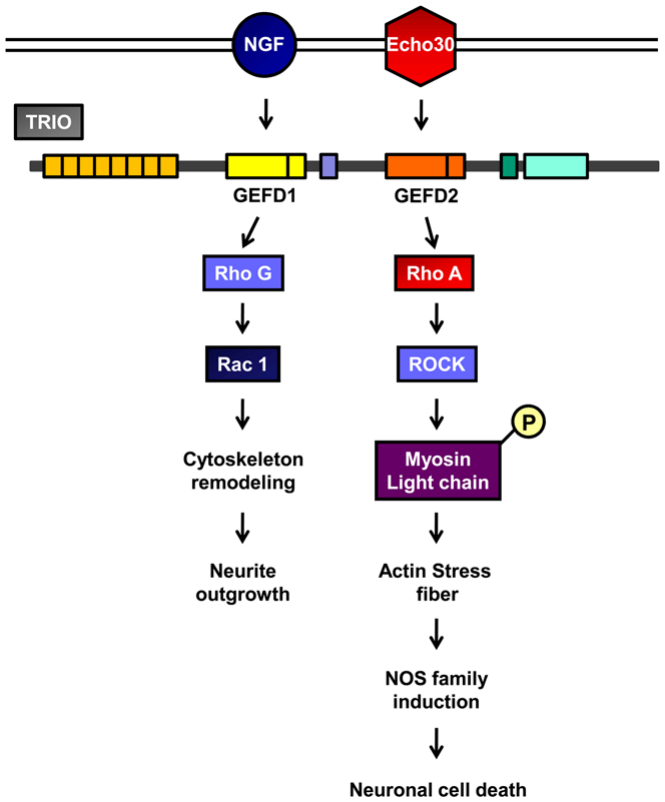
Schematic diagram of Echo30 induced TRIO activation on neuronal cell.

To examine the essential role of TrioGEFD2 in Echo30-induced neuronal cell death, SK-N-SH cells were pretreated with Y27632 in Echo30 infected SK-N-SH cells. As shown in Fig. 5A, B, the pretreatment of Y27632 partially prevented Echo30 infection induced cytopathic effect in SK-N-SH cells (Fig. 5A) and the inhibition of Echo30 infection reduced neuronal cell viability (Fig. 5B). Additionally, the pretreatment of Y27632 partially decreased the levels of sub-G1 phase (Fig. 5C) and RhoA signaling (Fig. 5D, 5E). These results suggest that Echo30 infection induced neuronal cell death through promoting cellular NO level and actin stress fiber formation via activation of TrioGEFD2 and RhoA signaling (Fig. 6).

## Discussion

Echo30 infection induces CNS disease [3], but the related pathogenic processes are poorly understood. Therefore, we tried to clarify the relationship between Echo30 infection and pathogenesis of CNS diseases with a clinical sample from a patient with aseptic meningitis. Preferentially, we performed the proteomic analysis using Echo30-infected SK-N-SH cells. As a result, we found interesting and significant changes in the protein expression level. Echo30-infected SK-N-SH cells significantly increase TRIO protein level. TRIO is a multifunctional protein in neuronal tissue, such as cell migration, proliferation and actin remodeling [17], [18]. TRIO protein contains two guanine nucleotide domains (GEFD1 and GEFD2) [14] and these domains are essential for cellular signaling but the function of these domains are distinct [14]. TRIO knock-out mice are embryonic-lethal [11], [31], which is required for cerebella development [11]. TRIO-deficient mice display severe phenotypes, including low survival rate, ataxia and multiple developmental defects of the cerebellum. TrioGEFD1 plays a vital role in neuronal cell migration, axon guidance and cerebellar development [11]. Nerve growth factor (NGF) binding the neurotrophin receptor leads to a neurite outgrowth through the activation of TrioGEFD1-RhoG signaling [13], [19], [32]. Initiation of GEFD1-RhoG signaling changes in gene expression, and rearrangements of the actin cytoskeleton are required for neurite outgrowth [13], [19], [32]. In Pheochromocytoma (PC12) cells, TRIO is the rate-limiting factor of NGF-induced neurite outgrowth by the activation of RhoG via GEFD1 [32]. The interaction between TRIO and Kindins220, the scaffolding molecule, has a central role in neurite outgrowth in PC12 cells and primary neurons [19]. However, no reports have examined the physiological role of TRIO protein in a neuronal cell death condition. The role of TrioGEFD2 is predicted to be related to the neuronal cell death induced by Echo30 infection. Our results provide evidence that the role of TRIO protein level increases in neuronal cells due to Echo30 infection. Echo30 infection leads to enhanced TRIO protein level, resulting in RhoA and RhoA targeted signaling activation, and contributing to SK-N-SH cell death. Activation of TrioGEFD2 induces RhoA and RhoA targeted signaling promotion [24], [25]. TrioGEFD2 expressing fibroblast cells increases RhoA-mediated actin stress fiber formation [14], [18]. In addition to the activation of ROCK, the downstream effector molecule of RhoA promotes filamentous (F) actin stress fiber formation by the phosphorylation of MLC [18], [33], [34], [35]. Actin stress fiber formation has been reported to be involved in cellular nitric oxide production [30]. Formation of actin stress fiber leads to an increased eNOS expression [30]. Nitric oxide (NO) is a signal messenger and pathological agent in neuronal cells [29], [36]. NO directly leads to the damage of neuronal cells, and excess NO is converted to peroxynitrite (ONOO^−^), which can injure brain tissue [37]. Furthermore, NO inhibits Rac-1 [36], downstream of TrioGEFD1-RhoG, and mediates cell migration [36]. We presumed an increase of cellular NO level inhibited to TrioGEFD1-RhoG signaling and activation of TrioGEFD2-RhoA signaling. Several recent studies have demonstrated that reactive oxygen species (ROS) can directly activate RhoA and induce stress fiber formation in rat2 fibroblast REF52 cells [38]. This report suggests that ROS-mediated RhoA activation is a potential regulatory mechanism in cells that can affect cytoskeletal dynamics [38]. Our study demonstrates that the activation of TrioGEFD2 is involved in cellular NO production through NOS family induction and participates in neuronal cell death. Pretreatment of ROCK inhibitor Y27632 significantly attenuated cellular NO production by Echo30 infection in SK-N-SH cells. These data demonstrate that Echo30 infection induces TrioGEFD2-RhoA signaling activation associated with enhanced cellular NO production in neuronal cells.

In conclusion, this study reveals that Echo30 infection enhances cellular NO production through TrioGEFD2activation, resulting in neuronal cell death. We expect the regulation of TrioGEFD2-RhoA signaling may represent a new therapeutic approach in treating Echo30- induced aseptic meningitis and encephalitis.

## Materials and Methods

### Cell line and Virus strain

The human neuroblastoma cell line SK-N-SH was obtained from the ATCC. The cells were cultured in MEM (Invitrogen, Carlsbad, CA, USA) supplemented with 10% FBS, 2 mM L-glutamine, 1 mM sodium pyruvate, 100 units/ml penicillin and 100 ug/ml streptomycin (Invitrogen, Carlsbad, CA, USA). Cells were maintained in 5% CO_2_ at 37°C incubator. We used stool specimen from Echovirus 30 (Echo30) infected aseptic meningitis patient in South Korea in 2008 (GeneBank: HQ833329.1) [39]. Stool suspensions were prepared by adding 1 g of stool sample, 10 ml PBS buffer and 1 ml chloroform, shaking vigorously in a mechanical shaker, and centrifuging at 1,500 rpm for 20 min at 4°C [40]. Viral RNA was extracted using magnetic beads (GM-AUTOPREP™ kit, Seoul, Korea), and the purified viral nucleic acid was processed using Freedom EVO (Tecan, Männedorf, Switzerland) [39]. Echovirus 30 detection was processed in the one step real-time RT-PCR and BLAST searching followed by sequencing [39], [40]. All experiments progress on SK-N-SH cells (1.5×10^5^ cells/ml) infected with Echo30 nurovalent strain at a M.O.I. of 1.

### Two-dimensional gel electrophoresis (2-DE) analysis

Non-infected SK-N-SH cells and Echo30 (nurovalent strain) infected cells were collected using a cell scraper. The cells were washed twice by suspending in ice-cold PBS followed by centrifugation at 10,000 rpm for 5 min. Suspended cells were in 2-DE lysis buffer containing 5 M urea, 2 M thiourea, and protease inhibitor cocktail (Roche, Indianapolis, IN, USA). The cell suspension was sonicated on ice five times with 25-W output, using an ultrasonic vibrator. The sonicated cells were centrifuged at 10,000 rpm for 30 min to remove DNA. For the first dimensional separation, 1 mg protein was focused on IPG strips (Immobiline DryStrip pH 3–10NL, 24 cm, GE Healthcare Life Science, Uppsala, Sweden) and a total of 80,000 Vh was applied. Prior to the second dimension, strips were incubated for 10 minutes in equilibration buffer (50 mM Tris-Cl, pH 6.8 containing 6 M urea, 2% SDS and 30% glycerol), first with 1% DTT and second with 2.5% iodoacetamide. For second dimensional separation, electrophoresis was performed 9–16% gradient polyacrylamide gels until the dye front reached the lower end of the gel. For quantifying the relative abundance of proteins, gels were stained with silver stained. Stained gels were scanned using a GS-710 imaging densitometer (Bio-Rad, Hercules, CA, USA) and quantitative analysis using PDQuest 2-D analysis software (version 7.0, Bio-Rad, Hercules, CA, USA). Quantity of each spot was normalized by total valid spot intensity. Protein spots were selected for the significant expression variation deviated over two fold in its expression level between Echo30 infected and non-infected SK-N-SH cells.

### Mass spectrophotometric analysis

Protein spots were excised from gels with a sterile scalped and placed into eppendorf tubes. Proteins were digested using trypsin (Promega, San Luis, Obispo, CA, USA) as previously described. For MALDI-TOF/TOF MS analysis, the tryptic peptides were concentrated by a POROS R2, Oligo R3 column (Applied Biosystems, Foster City, CA, USA). After washing the column with 70% acetonitrile, 100% acetonitrile and then 50 mM ammonium bicarbonate, samples were applied to the R2, R3 column and eluted with cyano-4-hydroxycinamic acid (CHCA) (Sigma, San Louis, MO, CA, USA) dissolved in 70% acetonitrile and 2% formic acid before MALDI-TOF/TOF MS analysis. Mass spectra were acquired on a 4800 Proteomic Analyzer (Applied Biosystems, Foster City, CA, USA) operated in MS/MS modes. Peptide fragmentation in MS/MS mode was by collision-induced dissociation (CID) using atmosphere as the collision gas. The instrument was operated in reflectron mode and calibrated using the 4800 calibration mixture (Applied Biosystems, Foster City, CA, USA) and each sample spectrum was additionally calibrated using trypsin autolysis peaks. The search program MASCOT (http://www.matrixscience.com) was used for protein identification by peptide mass fingerprinting with 0.1∼0.2 Da of mass tolerance for peptide ion (m/z) (Database: Swiss-Prot 56.9, 1 missed cleavage permitted).

### Immunoblots

For immunoblotting analysis, the cells were lysed in ice-cold RIPA lysis buffer containing 50 mM Tris-Hcl pH7.4, 150 mM NaCl, 1% Nonidet P-40, 1% sodium deoxycholate, 0.1% SDS, 1 mM EDTA, 1% SDS, 5 ug/ml aprotinin, 5 ug/ml leupeptin, 1 mM PMSF, 5 mM sodium fluoride and 5 mM sodium orthovanadate. BCA assay kit (Pierce, Rockford, IL, USA) was used as the measurement for protein concentration. The preparation of sample protein (30 ug) boiled for 5 min at 100°C and resolved by 5%, 10% or 15% SDS-PAGE and blotted on PVDF membrane with respective antibodies. The immunoblots were visualized by chemiluminescence using the ECL Western Blotting System (GE Healthcare, Buckinghamshire, England). Quantification of immunoblot band intensities was carried out using TINA 2.0 densitometric analytical system (Raytest, Straubenhardt, Germany). All protein measurements were divided by the corresponding GAPDH measurement for normalization and represented by arbitrary unit fold of control.

### RhoA activity assay

The cell lysate was prepared by using RIPA lysis buffer. The cell lysates were clarified by centrifugation at 12,000 rpm at 4°C for 10 min. Equal volumes of lysates were incubated with glutathione S-transferase (GST)-Rho binding domain (RBD, 30 ug) beads at 4°C for 3 hrs, and washed three times with Tris buffer containing 1% Triton X-100, 150 mM NaCl, 10 mM MgCl_2_, 10 ug/ml aprotinin, 10 ug/ml leupeptin, 1 mM PMSF, 1 mM sodium fluoride and 1 mM sodium orthovanadate. The bound RhoA proteins were detected by immunoblotting using a monoclonal antibody against RhoA (Santa Cruz biotechnology, Santa Cruz, CA, USA).

### Cell viability assay

WST-1 reagent (Roche, Indianapolis, IN, USA) was used for measuring cell viability. WST-1 determines the number of viable SK-N-SH cells remaining after Echo30 infection. SK-N-SH cells were cultured in 96-well plate and 1 M.O.I. Echo30 virus infection cultured for 48 hrs 5% CO_2_ at 37°C. 50 ul WST-1 reagent was next added, and the cells were incubated for 3 hrs. Then, the formazan product was determined using a microplate reader (Bio-Rad) absorbance at 450 nm.

### Flow cytometry analysis

For measurement of cell cycle distribution, non-infected or Echo30 infected SK-N-SH cells were incubated for 48 hrs. After trypsinization, approximately 10^7^ cells were collected by centrifugation at 8,000 rpm for 5 min. Cells were then washed in ice-cold PBS containing 1 mM EDTA, followed by resuspension and fixation in 80% ethanol for 6 hrs. Next, cells were washed with ice-cold PBS and resuspended in 500 ul of ice cold PBS containing 1 mM EDTA and 100 ug/ml RNase, followed by a 30 min incubation period at room temperature. Cellular DNA was then sustained by the addition of 50 ug/ml propidium iodide (PI), and cells were analyzed on a Beckman coulter FC500 (Beckman coulter, Indianapolis, IN, USA).

### Measure of Nitric oxide

The measured nitric oxide production was determined indirectly as its metabolic product (nitrite, nitrate), using a griess method. The griess reagent system (Invitrogen, Carlsbad, CA, USA) is based on the chemical diazotization reaction, which uses sulfanilic acid and N-(1-napthyl)ethylenediamine dihydrochloride. The SK-N-SH cells were cultured in the 96-well plate with 1 M.O.I. Echo30 virus for 48 hrs, after added 100 ul griess reagent reacted at room temperature for 15 min. The nitric oxide product was determined by using a microplate reader absorbance at 540 nm.

### Statistical Analysis

All the data are expressed as means ±S.E.M. Differences between the groups were determined by one way analysis of variance (ANOVA) using the SAS statistical analysis program (SAS). Duncan's multiple range test was performed to evaluate differences between the groups.
